# Losing the Ability in Activities of Daily Living in the Oldest Old: A Hierarchic Disability Scale from the Newcastle 85+ Study

**DOI:** 10.1371/journal.pone.0031665

**Published:** 2012-02-15

**Authors:** Andrew Kingston, Joanna Collerton, Karen Davies, John Bond, Louise Robinson, Carol Jagger

**Affiliations:** 1 Institute for Ageing and Health, Newcastle University, Newcastle upon Tyne, United Kingdom; 2 Institute for Health and Society, Newcastle University, Newcastle upon Tyne, United Kingdom; Marienhospital Herne - University of Bochum, Germany

## Abstract

**Objectives:**

To investigate the order in which 85 year olds develop difficulty in performing a wide range of daily activities covering basic personal care, household care and mobility.

**Design:**

Cross-sectional analysis of baseline data from a cohort study.

**Setting:**

Newcastle upon Tyne and North Tyneside, UK.

**Participants:**

Individuals born in 1921, registered with participating general practices.

**Measurements:**

Detailed health assessment including 17 activities of daily living related to basic personal care, household care and mobility. Questions were of the form ‘Can you …’ rather than ‘Do you…’ Principal Component Analysis (PCA) was used to confirm a single underlying dimension for the items and Mokken Scaling was used to determine a subsequent hierarchy. Validity of the hierarchical scale was assessed by its associations with known predictors of disability.

**Results:**

839 people within the Newcastle 85+ study for whom complete information was available on self-reported Activities of Daily Living (ADL). PCA confirmed a single underlying dimension; Mokken scaling confirmed a hierarchic scale where ‘Cutting toenails’ was the first item with which participants had difficulty and ‘feeding’ the last. The ordering of loss differed between men and women. Difficulty with ‘shopping’ and ‘heavy housework’ were reported earlier by women whilst men reported ‘walking 400 yards’ earlier. Items formed clusters corresponding to strength, balance, lower and upper body involvement and domains specifically required for balance and upper/lower limb functional integrity.

**Conclusion:**

This comprehensive investigation of ordering of ability in activities in 85 year olds will inform researchers and practitioners assessing older people for onset of disability and subsequent care needs.

## Introduction

Activities that are required to function independently in daily life, so called activities of daily living (ADLs), have long been seen as essential measures of disability in ageing studies and in clinical practice to assess care needs. When Basic (personal care) Activities of Daily Living (BADLs), for instance feeding, bathing, and toileting [1], are combined with Instrumental Activities of Daily Living (IADLs) which measure the ability to self-care within a household through activities such as shopping, cooking and doing housework [2], they better describe the spectrum of disability for a broader range of people [3]. In addition some researchers discriminate between BADLs, IADLs and mobility items such as walking a short distance, using steps and moving around the home, the latter comprising functional limitations (specific actions) rather than compound actions that form activities.

The hierarchical structure to the order in which loss of ability in both BADL and IADL items occurs has been confirmed by cross-sectional and longitudinal studies [3]–[7]. When both BADL and IADL items are considered together, difficulty with IADL items precedes difficulty with BADL items within the hierarchy [3]. The order of loss of ability to perform activities has also been classified in terms of four domains with each domain containing multiple activities that are similar in terms of their need for specific functional integrity combinations of dexterity, balance, strength and upper or lower extremity involvement [5]. For example the first abilities lost require manual dexterity and the last upper rather than lower limb control.

A number of issues remain unresolved in terms of the hierarchy of activities. Most studies have included only a small set of items, typically five or six and, if not selected to span the full range of disability, may result in floor effects. Few studies have investigated hierarchies separately for men and women, particularly important for IADLs such as cooking, which may be confounded with sex-specific household roles, so-called situational disability as opposed to ‘true’ functional disability [8]. In addition few studies have included large numbers of the oldest old, those aged 85 years and older, who represent the fastest growing section of the population [9].

The aims of this paper were to develop a hierarchical disability scale, using a much wider range of BADL, IADL and mobility items than previously employed which was appropriate for the men and women using cross-sectional (baseline) data from a birth cohort of over 800 85 year old participants in the Newcastle 85+ Study [10], [11]. In addition we aimed to validate the scale by examining its relationship with known predictors of disability.

We feel that further confirmation of the ordering of loss of BADLs and IADLs in this unselected single year birth cohort across a much wider range of activities, would assist researchers and clinicians in choosing subsets of activities that span the whole spectrum of disability and deepen understanding of the order in which older people lose functional capacity, thereby facilitating the design of more appropriate aids and appliances and the targeting of resources during the onset and progression of disability.

## Results

The study population for analysis comprised 839 of the health assessment sample (98.8%) this being participants who had both a health assessment with complete data on all BADL, IADL and mobility items, and a review of general practice records. Table 1 shows that women formed 62% (520) of the study population; 10.1% (85) of the study population resided in an institution and women were at least twice as likely to live in an institution compared with men (p = 0.004,OR = 2.1), 7.0% (58) had severe cognitive impairment (SMMSE 0–17) with no difference in prevalence between men and women (p = 0.54), and 25.5% (211) had three or more long standing illnesses, again with no difference between men and women (p = 0.15). Women were nearly twice as likely to take more than 12 seconds to complete the timed up-and-go compared to men (P<0.001, OR = 1.9). There was no statistically significant difference in the number of prescribed medications between men and women (p = 0.07) or in the number of diagnosed diseases they had (p = 0.10).

**Table 1:** Basic Descriptive Statistics of the Study Population by Sex.

	Men - %(n)	Women - %(n)	All - %(n)	p-value
**Living arrangements**				
Community	93.7 (299)	87.5 (455)	89.9 (754)	p = 0.0040
Institutions	6.3 (20)	12.5 (65)	10.1 (85)	
**Years of education**				
< = 9	60.8 (194)	64.4 (335)	63.1 (529)	p = 0.5350
10–11	24.1 (77)	21.2 (110)	22.3 (187)	
>11	15.1 (48)	14.4 (75)	14.7 (123)	
**MMSE**				
0–17	6.6 (21)	7.2 (37)	7.0 (58)	p = 0.0920
18–21	3.1 (10)	6.8 (35)	5.4 (45)	
22–25	18.2 (58)	14.7 (76)	16.1 (134)	
26–30	72.0 (229)	71.3 (368)	71.6 (597)	
**No. of longstanding illnesses**				
None	18.7 (59)	20.7 (106)	20.0 (165)	p = 0.1510
1	33.0 (104)	28.2 (144)	30.0 (248)	
2	26.4 (83)	23.3 (119)	24.5 (202)	
3+	21.9 (69)	27.8 (142)	25.5 (211)	
**Timed up and go test**				
≤12 seconds	43.5 (127)	28.5 (128)	34.4 (255)	p<0.0001
>12 seconds	56.5 (165)	71.5 (321)	65.6 (486)	
**Number of prescribed medications (median (IQR))**	6 (4–8)	6 (4–9)	6 (4–9)	p = 0.0723
**Simple disease count (median (IQR))**	4 (3–6)	5 (4–6)	5 (3–6)	p = 0.0999

Participants experienced most difficulty with cutting toenails, shopping and using steps and least with washing hands and face and feeding (Table 2). Women experienced significantly more difficulty than men with all items except dressing and light housework.

**Table 2:** Prevalence of ‘Difficulty’ in (I)ADL and Mobility Items - %(n).

(I)ADL or Mobility Item	Men	Women	All	OR (95% CI)§
Cutting Toenails	58.9 (188)	69.4 (361)	65.4 (549)	1.6 (1.2, 2.1)*
Shopping	38.2 (122)	63.1 (328)	53.6 (450)	2.8 (2.0, 3.7)*
Use Steps	38.9 (124)	54.4 (283)	48.5 (407)	1.9 (1.4, 2.5)*
Walk 400 Yards	39.5 (126)	53.1 (276)	47.9 (402)	1.7 (1.3, 2.3)*
Heavy Housework	30.1 (96)	56.7 (295)	46.6 (391)	3.0 (2.2, 4.1)*
Full Wash	25.4 (81)	38.8 (202)	33.7 (283)	1.9 (1.4, 2.6)*
Manage Money	19.7 (63)	27.1 (141)	24.3 (204)	1.5 (1.1, 2.2)*
Move Around House	17.6 (56)	25.6 (133)	22.5 (189)	1.6 (1.1, 2.3)*
Cooking a Hot Meal	18.2 (58)	25.2 (131)	22.5 (189)	1.5 (1.1, 2.2)*
Transfer from Chair	20.4 (65)	22.1 (115)	21.5 (180)	1.1 (0.8, 1.6)
Light Housework	16.9 (54)	21.7 (113)	19.9 (167)	1.4 (0.9, 2.0)
Transfer from Toilet	14.4 (46)	20.6 (107)	18.2 (153)	1.5 (1.0, 2.3)*
Manage Medications	14.4 (46)	20.0 (104)	17.9 (150)	1.5 (1.0, 2.2)*
Dressing	15.7 (50)	18.8 (98)	17.6 (148)	1.2 (0.8, 1.9)
Transfer from Bed	11.9 (38)	18.1 (94)	15.7 (132)	1.6 (1.1, 2.5)*
Wash Face & Hands	4.4 (14)	7.1 (37)	6.1 (51)	1.7 (0.9, 3.4)
Feeding	3.1 (10)	7.3 (38)	5.7 (48)	2.4 (1.2, 5.6)*

*Statistically significant gender difference at α = 0.05.

§ - Odds ratio: Women: Men.

PCA identified for men that one component (eigenvalue = 12.8) explained 75.4% of the variance with the second component (eigenvalue = 1.1) capturing only 6.7% further. Similar results were found for women, with the first component (eigenvalue = 11.0) capturing 71.8% of the variance and the second component (eigenvaue = 1.2) explaining a further 7.2%. This was further confirmed when men and women were analysed together; component 1 accounted for 76.2% of variation (eigenvalue 11.9); component 2 explained 6.2% of the variation (eigenvalue = 1.1). Whether men and women were analysed together or separately we found strong evidence of unidimensionality with component one always producing a far greater eigenvalue than component two and with approximate equal loading factors across all items (factor loadings all: minimum = 0.188, maximum = 0.262; men: minimum = 0.186, maximum = 0.271; women: minimum = 0.192, maximum = 0.268). However when men and women were analysed together, the second component indicated larger loading factors for the ‘manage money’ and ‘manage medication’ items, suggesting that this could be a dimension related to cognition, although this was less evident for men alone.

Mokken Scaling indicated that a hierarchy was present within the data and confirmed the unidimensionality conclusions of the PCA (Loevinger Scalability Co-efficient = 0.68). All items satisfied the assumption of single monotonicity thus suggesting that each item forms at distinct loci on a disability scale i.e. no items were measuring disability at exactly the same level. However, five items violated the assumption of double monotonicity: ‘transfer from chair’, ‘transfer from toilet’, ‘manage medications’, ‘move around the home’ and ‘manage money’ (women only) (Table 3). As the PCA indicated a possible second dimension related to cognition, loading on ‘managing money’ and ‘managing medications’, and since these items also failed the assumption of double monotonicity when men and women were analysed separately, the Mokken Scaling was repeated with these items removed. Removal of these items increased the strength of the hierarchical scale (Loevinger Scalability Coefficient ≥0.71) (Table 1), removed any further violation of assumptions and was significantly better than when cognition items were included. All analyses reported subsequently were calculated with the cognition items removed.

**Table 3:** Hierarchy of Loss of Ability in (I/B)ADL and Mobility Items Formed by Mokken Scaling.

Hierarchy Position	All	Men	Women
1 – Most ‘difficult’ (lost first)	Cutting Toenails	Cutting Toenails	Cutting Toenails
2	Shopping	Walk 400 Yards	Shopping
3	Use Steps/Stairs	Use Steps/Stairs	Heavy Housework
4	Walk 400 Yards	Shopping	Use Steps/Stairs
5	Heavy Housework	Heavy Housework	Walk 400 Yards
6	Wash all over	Full Wash	Full Wash
7	Manage Money	Transfer from Chair	Manage Money*
8	Cook a Hot Meal	Manage Money*	Move Around House*
9	Move Around House*	Cook a Hot Meal*	Cook a Hot Meal
10	Transfer from Chair*	Move Around House	Transfer from Chair
11	Light Housework	Light Housework	Light Housework
12	Transfer from Toilet*	Dressing	Transfer from Toilet*
13	Manage Medication*	Transfer from Toilet*	Manage Medication*
14	Dressing	Manage Medication*	Dressing
15	Transfer from Bed	Transfer from Bed	Transfer from Bed*
16	Wash Face & Hands	Wash Face & Hands	Feeding
17 – Least ‘difficult (lost last)’	Feeding	Feeding	Wash Face & Hands
Loevinger Scalability Coefficient	0.68	0.68	0.68
Loevinger Scalability Coefficient (with cognition items removed)	0.72	0.71	0.72

*Violated double monotonicity assumption (when cognition items were included).

**Figure 1:**
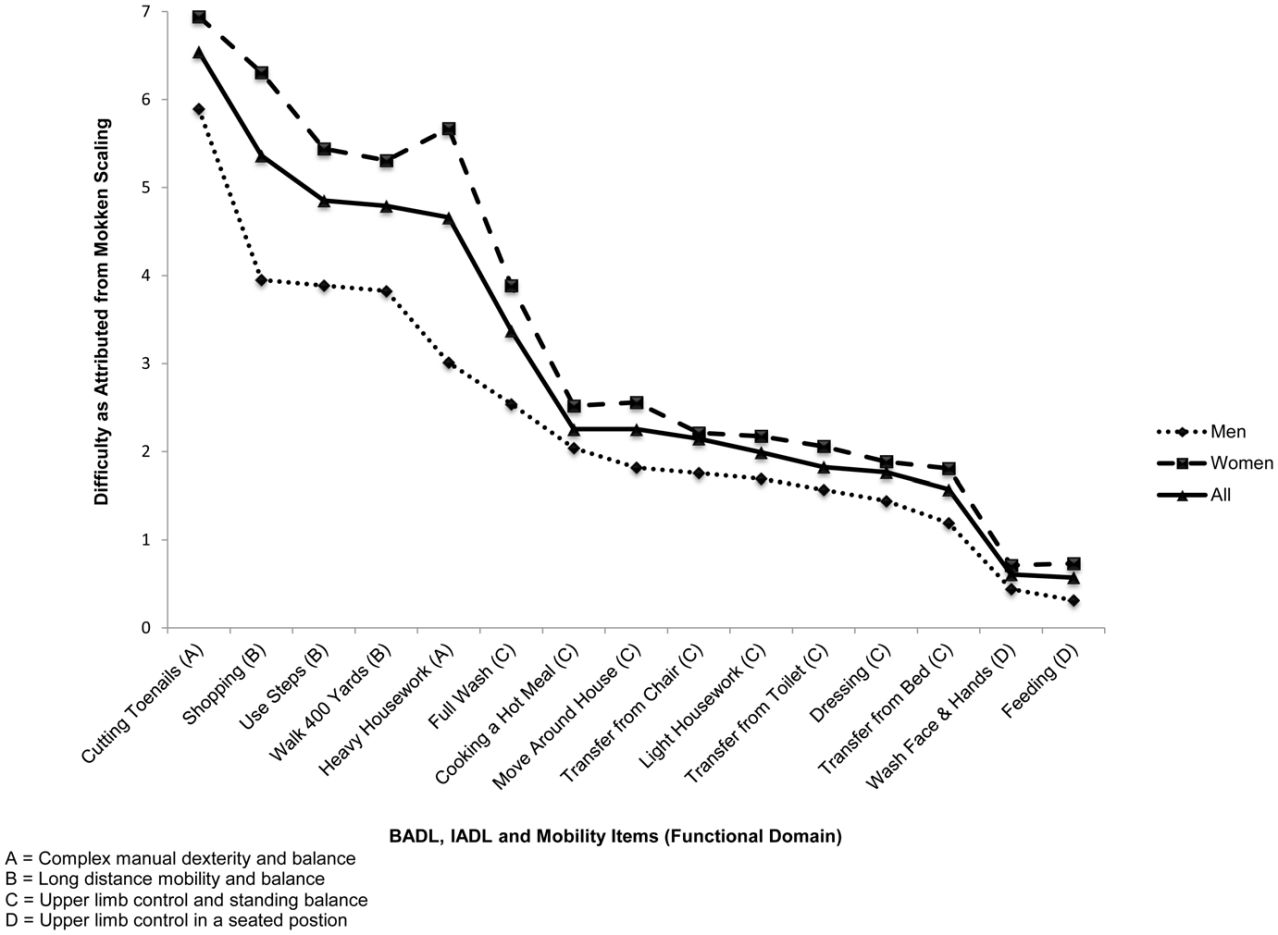
Relative Difficulty of BADL, IADL and Mobility Items (Domain of Disability [**5**]). Abbreviations: BADL – Basic Activities of Daily Living. IADL – Instrumental Activities of Daily Living.

For both men and women, ‘cutting toenails’ was the first activity with which participants had difficulty, and feeding was the last. The scaling algorithm constructed a numerical ordering for the items which indicated whether items had a tendency to be clustered together in terms of difficulty. When the scores of relative difficulty in performance of the items were plotted (Figure 1), clear areas of clustering of items was evident, corresponding with previously reported domains requiring particular combinations of lower and upper body strength combined with balance [5]; these being indicated in Figure 1 by; (A) – involving complex manual dexterity and balance; (B) – long distance mobility and balance; (C) – upper limb control and standing balance and (D) – upper limb control in a seated position.

The disability scale formed from assigning participants to the highest hierarchical position of the items with which a participant had difficulty, was highly correlated with the more usual scale formed by summing the number of items (out of 15) with which the participant had difficulty (Spearman's ρ = 0.94) and it had very strong internal consistency (Cronbach's α = 0.937). Further validation by comparison with known predictors of disability (Table 4) showed a significant association with all measures apart from education.

**Table 4:** Association of the Hierarchical Disability Scale with Known Predictors of Disability - %(n).

		Hierarchic Scale			
	None	1–5	6–10	11–15	p-value
**Living arrangements - %(n)**					
Community	98.8 (168)	99.6 (264)	89.4 (160)	72.0 (162)	p<0.001
Institutions	1.2 (2)	0.4 (1)	10.6 (19)	28.0 (63)	
**Years of education - %(n)**					
< = 9	61.8 (105)	63.8 (169)	62.0 (111)	64.0 (144)	
10–11	17.7 (30)	26.4 (70)	24.6 (44)	19.1 (43)	p = 0.8886
>11	20.6 (35)	9.8 (26)	13.4 (24)	16.9 (38)	
**MMSE - %(n)**					
0–17	87.1 (148)	81.5 (216)	66.5 (119)	51.8 (114)	
18–21	11.2 (19)	14.0 (37)	17.9 (32)	20.9 (46)	p<0.001
22–25	1.8 (3)	4.2 (11)	7.8 (14)	7.7 (17)	
26–30	0.0 (0)	0.4 (1)	7.8 (14)	19.6 (43)	
**No of longstanding illnesses - %(n)**					
None	40.6 (69)	18.6 (49)	15.3 (27)	9.3 (20)	
1	36.5 (62)	34.1 (90)	21.0 (37)	27.3 (59)	p<0.001
2	15.9 (27)	23.9 (63)	34.1 (60)	24.1 (52)	
3+	7.1 (12)	23.5 (62)	29.6 (52)	39.4 (85)	
**Timed up and go test - %(n)**					
≤12 seconds	66.7 (112)	39.2 (100)	17.5 (28)	9.5 (15)	p<0.001
>12 seconds	33.3 (56)	60.8 (155)	82.5 (132)	90.5 (143)	
**Number of prescribed medications (median (IQR))**	4 (2–7)	5 (3–8)	7 (5–10)	7 (5–10)	p<0.001
**Simple disease count (median(IQR))**	4 (3–5)	4 (4–6)	5 (4–6)	5 (4–6)	p<0.001

Exclusion of those residing in institutions had no effect on either the PCA or the Mokken Scaling procedure. Similarly, using a cut point of ‘needing help’ rather than ‘difficulty’ for the BADL, IADL and mobility items did not alter the conclusions.

## Methods

The Newcastle 85+ Study [10], [11] recruited a cohort of 1040 85 year olds from general practices in Newcastle and North Tyneside, UK. Eligible individuals were all those born in 1921 (aged around 85 at the time of recruitment) and who were permanently registered with a general practice in the study area. 83% (53/64) of general practices agreed to take part; those who declined were similar on practice size, the proportion who were training practices, National Health Service (NHS) Quality and Outcomes Framework score Index of Multiple Deprivation score (IMD) for 2004 to those agreeing. Participating general practitioners were asked to review patient lists prior to mail-out and to exclude only those individuals with end stage terminal illness (n = 11). All those who met these inclusion criteria were invited to participate (n = 1459), whether living at home or in an institution, and regardless of their state of health with recruitment and assessment taking place over a 17 month period during 2006–2007. A total of 358 people (24.5%) declined to participate, these being similar in terms of sex and deprivation to those who agreed to take part [10]. Study participants were assessed in their normal place of residence, including institutional care, by trained research nurses with a series of questionnaires, measurements, function tests, a blood test and a review of general practice records. Participants could decline parts of the assessments. Of the potential sample of 1040 people, 849 agreed to the health assessment and a review of general practice records; 188 to GP record review only and 3 agreed only to take part in the health assessment. Fewer females were in the health assessment plus record review group (62.0%, 526/849) than in the record review only group (72.3%, 136/188) (full details of the design of the study can be found elsewhere [10], [11]).

The present analysis was confined to those participants who had the health assessment since this was the only source of information on ADLs. During the health assessment, participants were asked if they were able to do the following activities: cut toenails, wash all over, transfer from a bed/toilet/chair, dress and undress, wash face and hands and self-feed (including cutting up food), shop for groceries, do light housework, do heavy housework, manage money, manage medications and prepare and cook a hot meal. In addition participants were asked three questions on mobility: get around in the house, go up and down stairs/steps, and walk at least 400 yards? Each question was framed as ‘can you’ rather than ‘do you’ to have greater capacity to assess true levels of disability [12] accounting for situational responses. Responses to all items were: I have no difficulty doing this by myself/ I have some difficulty doing this by myself/ I can only do this by myself if I use an aid or appliance/ I am unable to do this by myself, I need someone's help.

Socio-demographic information included sex, years of education, and institutional status, with additional variables including the number of longstanding illnesses; the number of prescribed medications (extracted from GP records); a disease count from the presence of 18 selected chronic diseases [10] and the timed up and go test [13] with a cut point that determines those with normal function as performing the test in 12 seconds or less [14]. Cognitive function was also measured by the Standardised Mini-Mental State Examination [15], [16] with severe cognitive impairment classified by a score of 17 or less out of 30, this cut point having high sensitivity for moderate and severe dementia [17].

Ethnical approval was obtained from Newcastle & North Tyneside Local Research Ethics Committee One and informed written consent was obtained from all participants.

To determine whether the BADL, IADL and mobility items formed a single dimension we used Principal Component Analysis (PCA) based on the polychoric correlations between items and with whole ordinal scales. The number of dimensions was determined using Kaiser's Criterion, including only eigenvalues greater than one [18]. Having identified a single dimension, we dichotomised the items using a cut point of no difficulty/some difficulty (from a four category response of ‘no difficulty’, ‘some difficulty’, ‘only with and aid’ and ‘unable to do this’). We then used Mokken Scaling to verify the unidimensionality and to determine the hierarchy from the Loevinger Scalability Coefficient (H) [19] with values of H between 0.3–0.39 being taken to suggest a weak Mokken scale; between 0.4–0.49 an acceptable Mokken scale and greater than 0.5 a strong Mokken scale [20]. Items were deleted from the scale if they did not satisfy the assumption of single monotonicity (each item forms at a distinct loci on a scale of decreasing difficulty) and double monotonicity (the Item Characteristic Curves are non-overlapping). As Mokken scaling uses multiple tests on the data a Bonferroni correction was implemented to reduce the type I error. A scoring system was formed based on the highest item in the hierarchy with which the participant had difficulty (a score of 1 being low indicating difficulty with the first (most difficult) item in the hierarchy and a score of 17 being the highest indicating difficulty with then last (least difficult) item in the scale). Participants having no difficulty with all items in the scale were assigned a score of zero. Cronbach's alpha [21] was used to assess the internal consistency of the scale with values close to one suggesting a strong scale and values close to zero indicative of poor internal consistency. We constructed a disability scale corresponding to the highest hierarchical position of the items with which the participant had difficulty. After separation into four categories; difficulty with no items, difficulty with 1–5 items, difficulty with 6–10 items and difficulty with 11–15 items (to allow for nonlinear associations) the scale was validated against known predictors of disability [22].

Separate analyses were carried out for men and women. Sensitivity analyses were undertaken excluding the participants living in institutions and using the alternative cut-point for performance of no help required/help required. All analyses were carried out in Stata 10.1 [StataCorp. 2009. Statistical Software: Release 10.1. College Station, TX: Stata] with statistical significance at α = 0.05.

## Discussion

We found a strong hierarchical ordering to loss of ability in a wide range of basic and instrumental activities of daily living and the items measuring mobility in an unselected population aged 85 years in 2006. ‘Cutting toenails’ was the first item with which participants found difficulty and ‘washing hands and face’ and ‘feeding’ the last items. The ordering of the items in our hierarchic scale confirms previous studies using cross-sectional [3], [5], [6], [23] and longitudinal [4], [7] data, but which are now 10–20 years and were based on a more restricted set of items predominantly in the younger old. Thus our study adds considerably to the evidence that the order of loss of activities does not vary with age. Sex differences were evident from our single birth year cohort; not only were women more likely to report difficulty with each activity than were men but we also found that the ordering of loss differed between men and women of the same age with women reporting more difficulty with activities requiring strength (‘shopping’ and ‘heavy housework’) whilst men were earlier in reporting difficulty walking. Moreover the ordering and our conclusions were unchanged if inability to perform was defined as requiring the help of another person rather than the more unbiased having difficulty performing alone. However measuring disability by the requirement for ‘help’ may depend on the availability of help which may therefore bias results [24] and thus our primary measure based on ‘difficulty’ adds strength to our study.

Previous research has indicated that disability in later life appears to progress with difficulty in IADLs preceding that with BADLs [4], [6], [7], [23]. Though this was broadly true in our analysis; there was overlap in the ordering of IADLs and BADLs and our ordering was much more consistent with the domains of disability defined by Ferrucci et al [5] which combine IADL, BADL and mobility items requiring similar underlying impairments. They defined the first domain in which difficulty would be as activities requiring complex manual dexterity coupled with balance such as ‘cutting toenails’ and ‘heavy housework’. Activities in the next domain require balance and involve the capacity to walk long distances, the equivalent activities in our study being ‘shopping’, ‘use steps’, ‘walk 400 yards’, ‘full wash’, ‘cooking a hot meal’, ‘light housework’. The third domain in decreasing difficulty contained activities requiring standing balance and good upper limb control; ‘move around the home’, ‘transfer from chair’, ‘toilet’, ‘dressing’ and ‘transfer from bed’. The final domain, and the easiest to perform, related to good upper limb control when in a seated position; ‘washing face and hands’ and ‘feeding’. Only two items appeared out of step between our hierarchy and Ferrucci's four domains; ‘heavy housework’ and ‘light housework’ and it may be that the perceived meanings and the nature of these tasks have changed more over time than for other activities.

Our disability scale formed from the hierarchy performed well when examined alongside known predictors of disability. The ordering of loss of activities is of potential use to others selecting activities to measure a range of severity of disability both in the research and clinical setting. We collected information from over 800 older people aged 85 years of age on their situational disability (questions being framed as ‘can you’ rather than ‘do you’) from 17 IADLs, BADLs and mobility items, a much larger number than previous studies and with minimal missing data. The Newcastle 85+ Study has a broad range of health measures and is representative of the larger population of older people in Newcastle upon Tyne [10].

The main limitation of our study is the cross-sectional nature of the data although our results were in agreement with the previous longitudinal studies [4], [7] Nevertheless, unlike other studies, our population came from a single birth cohort with a high response rate and included those in institutions. Thus the sex difference we found in the order of loss of activities was not due to the greater average age of women compared to men in general older populations. Inclusion of those in institutions where there is a high prevalence of dementia may be viewed as a limitation. We had a small proportion of our sample (n = 71, 8.5%) who had a diagnosis of dementia and these participants may lose ability to perform activities independently in a different order to those without dementia, for instance they may be able to walk 400 yards but may not be able to dress themselves. However, we repeated all analyses excluding those in institutions and the conclusions were unchanged. Nevertheless, when we originally included items more dependent on complex cognitive ability than physical ability (‘managing money’ and ‘managing medication’) we found that they did not satisfy all of the underlying assumptions of the scaling method and their subsequent exclusion strengthened the scale formed although the remaining items may be viewed as being more ‘physical’.

Results of this analysis could therefore provide information to help identify older people at risk of functional decline and for the allocation, and prioritisation of, community services and social support to enable independent living for as long as possible. We found in terms of the ranking of difficulty that a number of items were ranked similarly suggesting that if these items alone were selected for inclusion in a disability scale, or indeed for assessment purposes, then the range of severity would be limited. ‘Cutting toenails’ and ‘shopping’ were the items with which our population most commonly reported difficulty. The former requires good balance and manual dexterity whilst the latter requires upper body strength and mobility. Physical activity programmes to delay the onset of disability should perhaps focus on exercises to improve these functions; in addition our results could support an argument for the essential core provision of specific services such as chiropody within the community care, as opposed to the current limited provision.
